# Challenging human somatic testicular cell reassembly by protein kinase inhibition –setting up a functional *in vitro* test system

**DOI:** 10.1038/s41598-020-65924-y

**Published:** 2020-06-02

**Authors:** M. Mincheva, J. Wistuba, C. Brenker, S. Schlatt

**Affiliations:** Centre of Reproductive Medicine and Andrology, University Hospital of Münster, Albert-Schweitzer Campus 1, 48149 Münster, Germany

**Keywords:** Cell biology, Developmental biology

## Abstract

Signalling pathways and cellular interactions defining initial processes of testis morphogenesis, i.e. cord formation, are poorly understood. *In vitro* cell-based systems modelling cord formation can be utilised as platforms to interrogate processes of tubulogenesis. We aimed at testing our established cord formation *in vitro* model using adult human testicular cells as a quantitative assay that can facilitate future studies on cord morphogenesis. We challenged the responsiveness of our system with a broad-spectrum protein kinase inhibitor, K252a. Cultured testicular cells were treated with various K252a concentrations under constant exposure and compound withdrawal. To quantify cell reaggregation changes, we performed computer-assisted phase-contrast image analysis of aggregate size and number. Cell reaggregation was analysed in detail by categorisation of aggregates into size groups and accounting for changes in aggregate number per size category. We found a dose-related disturbance of testicular cell reaggregation. K252a decreased aggregate size (IC50 of 203.3 nM) and reduced the large aggregate numbers. Video recordings revealed that treatment with K252a at a concentration above IC50 interfered with aggregate coalescence into cords. Short-term exposure and compound wash-out induced irreversible decrease in large aggregates. We propose our *in vitro* model as a functional platform to quantitatively investigate seminiferous tubulogenesis under pharmacological impact.

## Introduction

The initial process during male sex differentiation towards testis formation is testicular cord formation, taking place during embryonic development. Although the gross morphological events resulting in testis cords have been described, the processes and cellular interactions that define cord morphogenesis are poorly understood^1–4^. Rodent embryonic testis culture experiments showed that pre-Sertoli cell aggregation and migration of somatic cells, such as pre-peritubular cells, are relevant steps during cord formation^1,2,5,6^. These processes are regulated by paracrine growth factors which act as chemotactic agents that orchestrate cell migration and cell-cell interactions^2,5,6^. However, detailed information on somatic testis cell interplay is missing. Thus, an *in vitro* system resembling these processes might lead to better understanding of the developmental sequences and of causes for testis-related diseases and infertility, many of which originate in early development^7^.

In this regard, cell suspension-based *in vitro* culture systems have the advantage over tissue explant-based culture approaches because they facilitate the determination of cellular interactions and pathways regulating testicular tubulogenesis^8^. Animal studies using xeno-transplantation in rodents and primates demonstrated the intrinsic capacity of enzymatically dispersed testicular cells to re-organise into seminiferous cords *in vitro* and *in vivo*^9–15^. These *in vitro* cellular models of cord morphogenesis proved to be suitable to address scientific questions dealing with dynamic cellular behaviour and role of chemotactic agents during cord formation.

Recently, we established an *in vitro* system using human primary testicular cells to model cord morphogenesis^16^. We demonstrated that dispersed testicular cells are capable of reorganising spontaneously into cord-like structures by cellular aggregation, compaction and coalescence of the reassembled aggregates. Further, employing histology, immunohistochemistry and time-lapse microscopy analyses, we confirmed that Sertoli and peritubular were the somatic testicular cells involved in testicular cell reaggregation into cord-like structures.

Once established, the purpose of this study was to determine the responsiveness of our *in vitro* system to manipulation of cellular behaviour following pharmacological challenge. Exemplarily, we employed a broad-spectrum protein kinase inhibitor, K252a^17^ that was previously reported to perturb cord formation in rodent *in vitro* studies^18–20^. Our objective was to test our system in a functional challenge and to define measurable endpoints to quantitatively assess the degree of interference with cellular reassembly. As a proof of principle, we demonstrate that our model system can be used as a functional assay with quantitative endpoints that could be developed in a tool to interrogate processes of tubulogenesis in future studies.

## Results

### K252a interferes with *in vitro* cell reassembly

**Figure 1:**
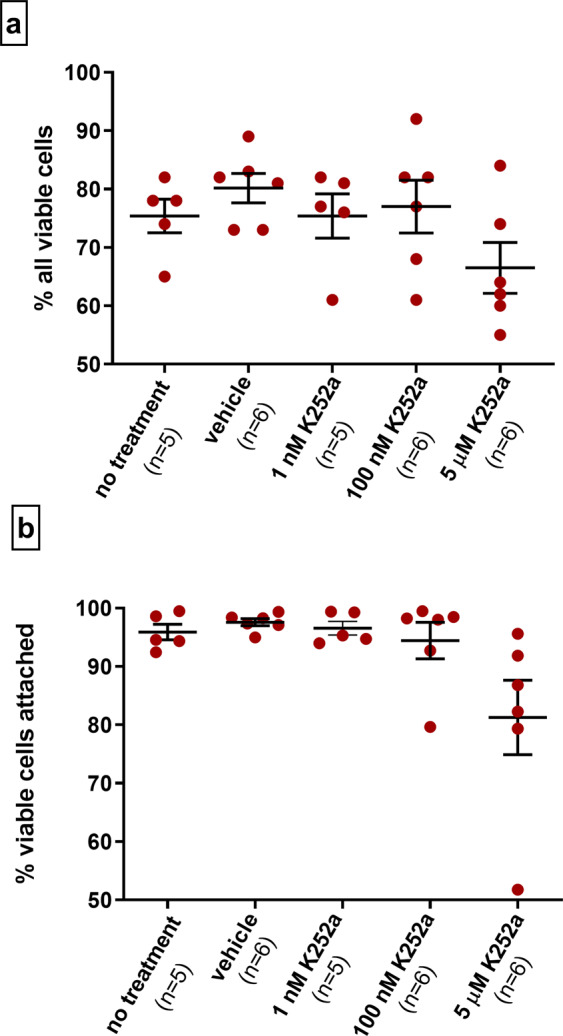
Cell viability 48 hours after plating in controls and K252a treated groups. Total cell viability and cell viability in the attached fraction following exposure to K252a (1 nM, 100 nM, 5 µM) do not differ from that of control (no treatment). Statistical test: Kruskal-Wallis with Dunn’s multiple comparison post hoc test versus no treatment control. Mean ± SEM is indicated. Numbers of biological experiments are shown in brackets. (**a**) Proportion of all viable cells from each experiment is expressed as a percentage of all cells (dead and alive) in floating and attached cellular fractions. (**b**) Proportion of viable cells in attached fraction from each experiment is expressed as a percentage of all viable cells (in both floating and attached fractions).

We first determined whether protein kinase inhibitor K252a influenced cell viability and attachment in the first 48 hours following cell seeding. The proportion of all viable cells amongst K252a concentration range did not differ when compared to that of no treatment control (Fig. 1a). Similarly, the proportion of attached live cells was comparable to that of no treatment control (Fig. 1b). This indicates, that K252a did not affect cell viability during the experiments and suggests that its presence did not disturb cell attachment which had already occurred 48 hours after seeding.

**Figure 2:**
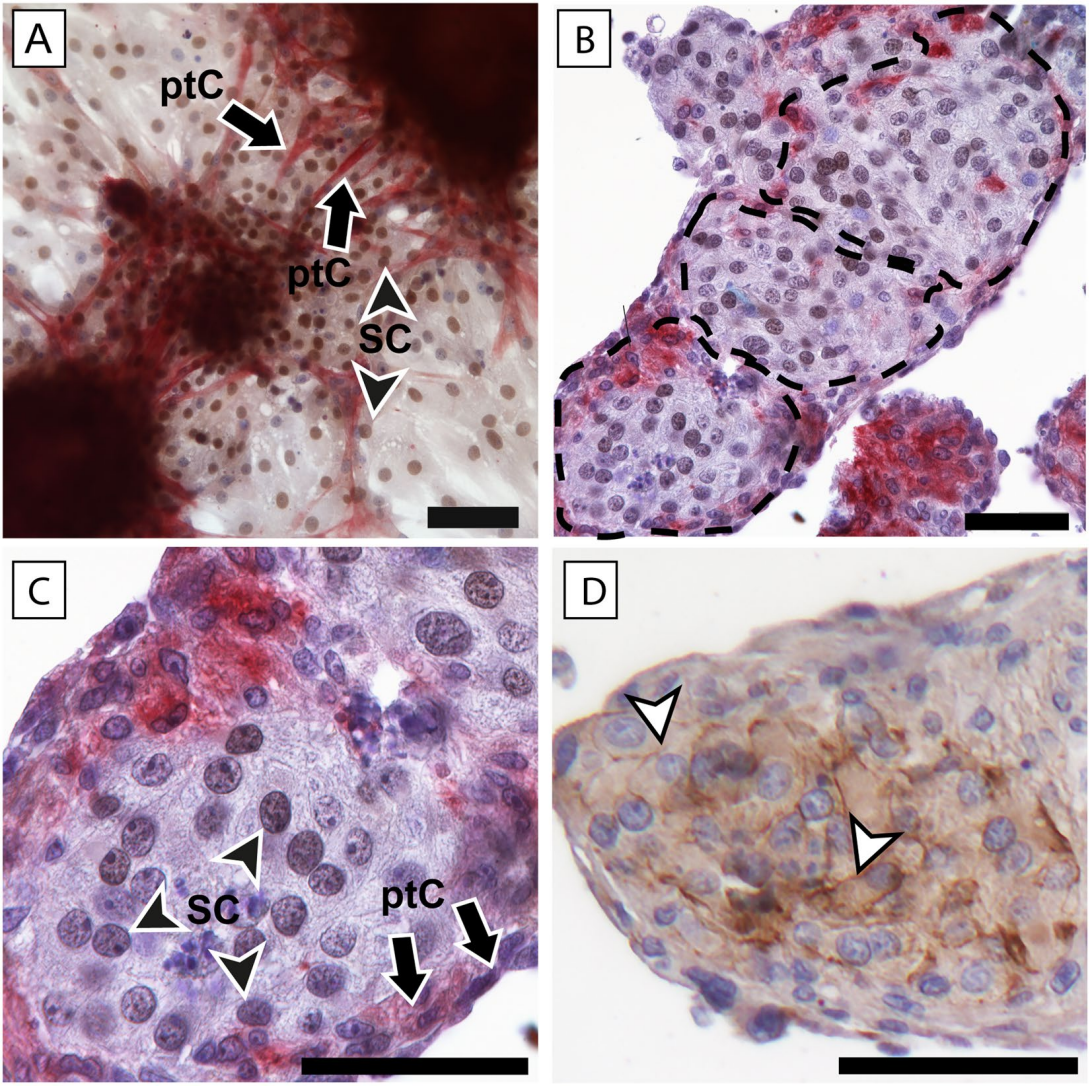
Immunohistochemical characterisation of cytoarchitecture of *in vitro* re-assembled cord-like structures by protein localisation of markers for Sertoli and peritubular cells and Sertoli-Sertoli cell junctions. Immunohistochemical staining was performed on cultured cells from at least three different patients’ samples. Enzymatically isolated cells were cultured in complete medium without K252a compound. (**A**–**C**) Double staining for *SRY*-related high mobility box 9-(SOX9) (with diaminobenzidine (DAB), brown colour) and α-smooth muscle actin (αSMA) (with fast red, red colour). (**D**) Single staining on a consecutive section of the same aggregate (in B) for tight junction protein-1 (ZO-1) (with DAB). (**A**) Representative image of stained whole aggregates formed after 4 days in culture. Alpha-SMA-positive peritubular cells, ptC (arrows) protrude from coalescing aggregates comprised by SOX9-positive Sertoli cells, SC (black arrowheads). (**B**,**D**) Elongated multi-cellular cord-like structures following aggregate coalescence. Representative image showing structured Sertoli and peritubular cell arrangements in consecutive cross-sections of an cord-like structure. (**B**,**C**) Typical spatial arrangement (dotted areas in B) of Sertoli and peritubular cells in *in vitro* formed cords. Strands of flattened peritubular cells (arrows) surround clusters of Sertoli cells (black arrowheads) (C is the magnified proximal dotted area in B). (**D**) ZO-1-positive Sertoli cells (D is the magnified proximal dotted area in B) re-established junctional connections (white arrowheads). Scale bars = 50 µm.

We next analysed morphologically the effects of K252a on cellular reaggregation in our *in vitro* system. Initially, we validated the structured reaggregation of Sertoli and peritubular cells in cord-like structures by immunohistochemical analyses of control cultures, not treated –with K252a (Fig. 2). We confirmed that coalescing round aggregates were connected by alpha-smooth muscle actin (αSMA) positive peritubular cells, and a preceding formation of elongated cord-like structures within a week of *in vitro* culture (Fig. 2A). Further analysis of cord-like structures cross-sections revealed their spatial cytoarchitecture - Sertoli cells were located centrally and peritubular cells arranged at the periphery (Fig. 2B,C). Sertoli cells also expressed proteins of the blood-testis barrier (detection of Zonula occludens, ZO-1, Fig. 2D).

**Figure 3:**
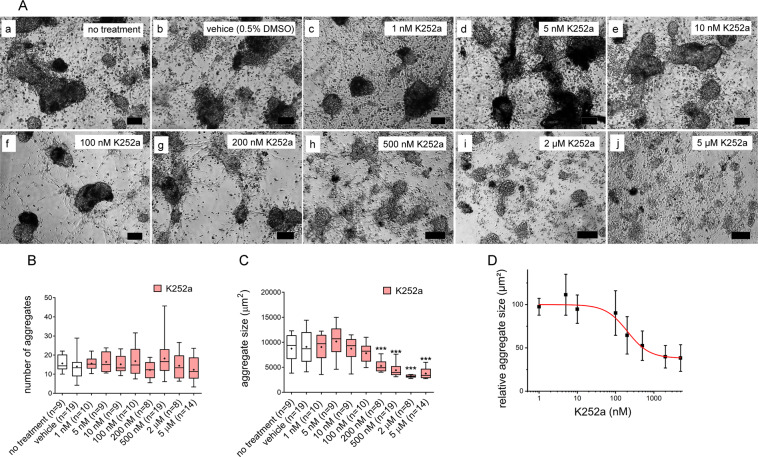
K252a disturbs reaggregation of human testicular cells. Enzymatically dispersed adult human testicular cells were cultured under constant exposure to K252a (1 nM–5 µM) for 3–5 days. (**A**) Representative phase-contrast micrographs revealing the inhibitory effects of K252a on cell reaggregation. Cells reassembled in compact aggregates in control groups, no treatment and vehicle (a and b, respectively). Treatment with K252a at 200 nM, 500 nM, 2 µM and 5 µM induced partial inhibition of cellular reassembly (g–j). Exposure to K252a at 1 nM, 5 nM, 10 nM and 100 nM did not interfere with cellular reaggregation (c–f). Scale bars equal 100 µm. (**B**) K252a did not affect the number of formed aggregates (Kruskal-Wallis and Dunn’s multiple comparison versus vehicle control). (**C**) Exposure to K252a at 200 nM–5 µM decreased median aggregate size (one-way ANOVA, Dunnett’s multiple comparison versus vehicle control, ***p < 0.0001). Data in (**B**,**C**) are shown as median and interquartile range, whiskers: min-max, +: mean. (**D**) Dataset from (**C**) was used to calculate IC50 ± standard error of the fit for median aggregate size (203.3 ± 47.8 nM).

Challenging the *in vitro* system with K252a showed that testicular cell reaggregation in cord-like structures was dose-dependently disturbed after exposure to K252a (Fig. 3A). While multi-cellular compact cord-like structures were observed in controls (no treatment and vehicle, Fig. 3A a,b), in cultures treated with 500 nM–5 µM K252a cells reassembled at maximum to small dispersed aggregates (Fig. 3A h-j).

### K252a affects aggregate size of *in vitro* reassembled cells

To account for the observed inhibitory effects of K252a on cellular reaggregation, we quantified aggregate number and size under the experimental conditions. Initially, we validated our quantitative approach by calculating intra-assay CV for aggregate number and size in control groups (no treatment and vehicle). Intra-assay CV for no treatment control ranged from 2.5 to 11.9% (aggregate size) and 2.6 to 10% (aggregate number), while for vehicle control the values ranged from 1.3 to 15.9% and from 0.8 to 14.6%, respectively. These CV values confirmed that the culture approach could be performed with high reproducibility and reliability. Subsequently, we compared aggregate number and size in treatment groups to vehicle control (Fig. 3B,C). While the number of aggregates was similar for all K252a concentrations (Fig. 3B), treatment with K252a in the range of 200 nM–5 µM significantly decreased median aggregate size (Fig. 3C). The IC50 for the inhibitory effect of K252a on median aggregate size was 203.3 ± 47.8 nM (Fig. 3D).

### K252a prevents cell reassembly in large aggregates and those effects are irreversible

**Figure 4:**
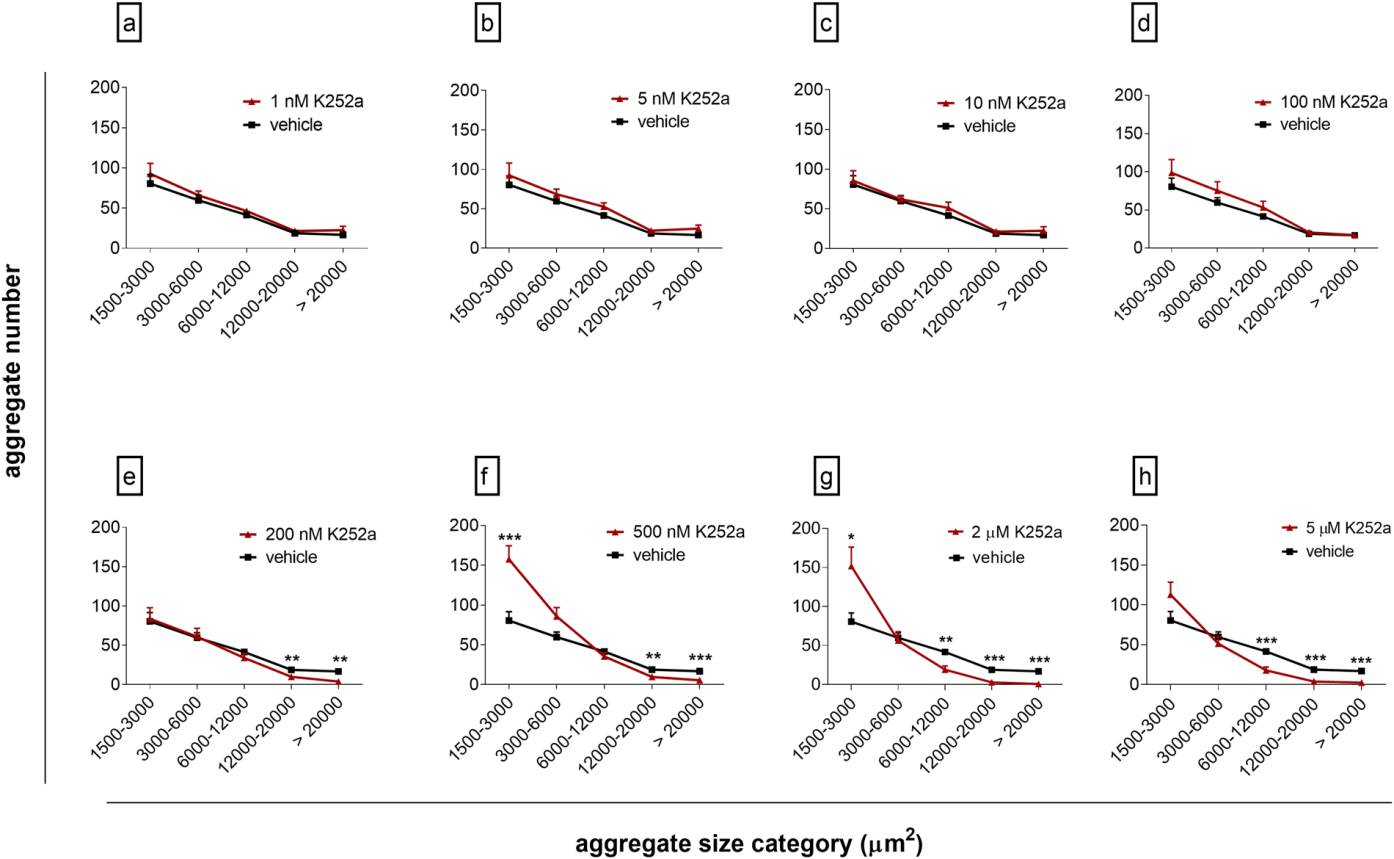
Constant exposure to K252a influences aggregate number across size categories. Human testicular cells were cultured for 3–5 days under constant exposure to K252a (1 nM–5 µM). K252a in the rage of 1–100 nM (**a**–**d**) did not evoke change in aggregate number across size categories. Treatment with K252a at 200 nM–5 µM (**e**–**h**) decreased number of aggregates larger than 6000 µm^2^ and increased aggregate numbers in 1500–3000 µm^2^ size category. Data are shown as mean (+SEM), Mann-Whitney U test versus vehicle control, *p < 0.05, **p < 0.01, ***p < 0.001.

Since we observed a difference in aggregate size between treatment groups and vehicle control, we evaluated in further detail the effect of K252a on cell reassembly by defining a range of aggregate size categories (Supplementary Fig. 1). We then quantified the aggregate numbers from each size category for treatment groups and vehicle control (Fig. 4). Treatment with 200 nM–5 µM K252a significantly reduced formation of large aggregates (>6000 µm^2^; Fig. 4e–h), while aggregate number in the smallest size category increased under exposure to 2 µM and 500 nM K252a (Fig. 4f,g).

We also determined whether initial short-term exposure to K252a followed by compound wash-out would rescue cellular reaggregation (Supplementary Fig. 2). Withdrawal experiments confirmed that decrease in the number of large aggregates was irreversible when cells were exposed for 2 days to 2 µM and 500 nM K252a (Supplementary Fig. 2c,d). However, inhibitory effects of 200 nM K252a on aggregate numbers were rescued after withdrawal (Supplementary Fig. 2b).

### K252a disturbs dynamics of *in vitro* cord-like structure formation

To assess the effects of high K252a concentrations (5 µM and 500 nM) on dynamics of cellular reaggregation and cord-like structure formation, we recorded time-lapse videos in the first 4 days of exposure (16-44 h). In both, no treatment (Supplementary Video 1) and vehicle (Supplementary Video 2) controls, cells reassembled in aggregates that further compacted and coalesced by cellular motions. Exposure to 500 nM K252a, however, interfered with coalescence of adjacent aggregates and with formation of compact cord-like structures (Supplementary Video 3). When treated with 5 µM K252a, cells reassembled into loose aggregates that remained still and rigid, with no evident cellular movements or protrusions from the aggregates (Supplementary Video 4).

### *In vitro* cord-like structure formation is persistently disturbed by constant exposure to K252a

**Figure 5:**
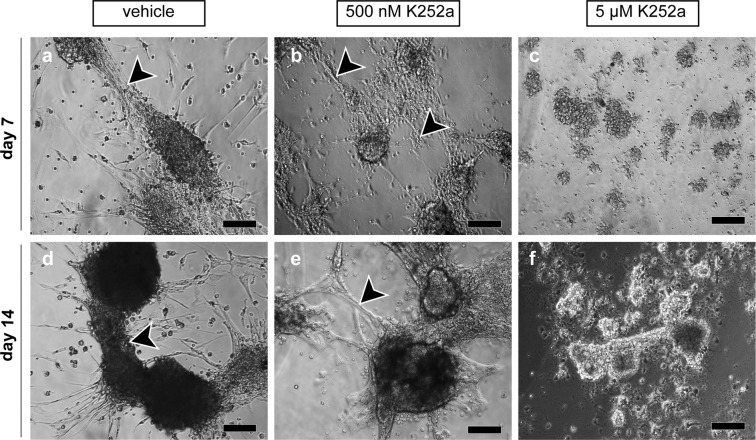
Two-week follow-up of the influence of K252a on *in vitro* testicular cord-like structure formation. Adult human testicular cells were cultured under constant exposure to K252a at 500 nM and 5 µM, and vehicle control for two weeks. Representative phase-contrast micrographs revealing the effect of K252a on *in vitro* cord-like structure formation captured at 7 and 14 days of testicular cell culture. Bundles of elongated cells connected neighbouring cords (arrowheads in **a**,**d**). Dispersed single elongated cells protruded from aggregates and interconnected adjacent aggregates (arrowheads in **b**,**e**). Exposure to 5 µM K252a completely inhibited protrusion of elongated cells from the aggregates (**c**,**f**) and formation of cord-like structures. Scale bars equal 100 µm.

Since large aggregate numbers were significantly decreased following constant exposure to 200 nM–5 µM K252a (Fig. 3C), we performed a two-week follow up on the effect of high K252a concentrations (5 µM and 500 nM) on aggregate morphological changes (Fig. 5). Aggregates in vehicle control had already interconnected via bundles of elongated cells and initiated coalescence at day 7. These morphological changes progressed further towards the formation of larger and compact aggregates by day 14 (Fig. 5a,d), while exposure to 5 µM K252a completely inhibited aggregate connections (Fig. 5c,f). Treatment with 500 nM K252a affected aggregate interconnection such that only single dispersed elongated cells extended from the aggregates and did not form further dense bundles as in vehicle controls (Fig. 5b,e).

## Discussion

*In vitro* testicular cell culture models of seminiferous tubulogenesis can be applied in a versatile fashion to understand signalling pathways and processes of seminiferous tubulogenesis^10–13,19^. In this proof of principle study, we demonstrated that our recently described primary human testicular cell culture system^16^ is responsive to pharmacological challenge with protein kinase inhibitor K252a with expected inhibitory effects on cord formation. We validated the model as a quantitative assay with robust endpoints that enable analysis of *in vitro* cellular reaggregation and cord-like structure formation patterns.

Our results reveal a dose-dependent disturbance of cell reassembly and a shift from larger to smaller aggregates at IC50 of 203.3 nM for K252a. Concordant to our data, a study using a cell-based assay with immature rat testicular cells demonstrated disruption of cord formation by characterising inhibition of reaggregation with parameters similar to ours (i.e. aggregate diameter and distance between aggregates)^19^. However, in contrast to complete inhibition of aggregate formation observed at 5 nM K252a in Gassei *et al*.^19^, testicular cells in our system reassembled, albeit in significantly smaller aggregates, across the entire K252a concentration range applied. Other studies exposing entire undifferentiated rat gonadal primordia to 100 nM K252a described a complete inhibition of testis cord formation^18,21^. It can be argued that this discrepancy is due to that those studies used entire organs, where tissue integrity and cellular associations are preserved. Another *in vitro* study using second trimester human foetal testes showed altered number and proliferation of testicular somatic cells, i.e. Sertoli and peritubular cells, when gonads were treated with 100 nM K252a for 48 hours^20^. It is commonly acknowledged that adult testicular somatic cells are non-proliferative^22^. Although we have not assessed cell proliferation in our study, we did not observe change in cell viability after exposure to K252a for 48 hours. Nevertheless, it is known that progression through different stages of foetal and postnatal testis tubulogenesis is marked by changes in proliferation and protein expression in somatic testicular cells^23–25^.

It is also plausible that there are species- and age-related differences in specific signalling pathways between human and rodents. As an example, studies addressing protein and gene expression of neurotrophins in testis have shown species- and cell type-specific expression of those factors and the expression was also age-dependent^18,26–29^. It was assumed that neurotrophins and their receptors are involved in seminiferous tubulogenesis and some of these studies used K252a at 100 nM to functionally challenge cord formation^18,20,21^. However, apart from neurotrophin receptor tyrosine kinases, K252a targets several protein kinases in the IC50 range between 3 and 25 nM^17,30,31^. Given this general ability to interfere with tubulogenesis at various levels and targets, we did choose this broad spectrum inhibitor as it was perfectly suited to test our system and to identify valid parameters that reflect the impact of this pharmacological challenge. However, applying an array of selective inhibitors at a range of concentrations to define dose-response curves will be required to dissect cellular mechanisms of testis tubulogenesis^32^. Furthermore, since no IC50 values were calculated in the aforementioned studies the conclusions that can be drawn may be limited^18,21,33^.

Tissue or organ culture approaches enable investigation of cell signalling pathways of seminiferous tubulogenesis^34–36^. However they are limited by low-throughput and do not allow simultaneous exploration of different conditions. Here, we validated the application of single cell suspensions to explore *in vitro* testicular cord formation. We propose that our cell-based assay with quantitative endpoints relevant to cell aggregation can facilitate future studies that address stage-timed proliferation, gene and protein molecular analyses for specific cellular mechanisms and processes. To characterise the role of cellular migration and contractility during cord morphogenesis^1,10^, this cell culture system can – in more specific applications - be coupled with advanced analytical techniques such as traction force microscopy^37–39^. It may then be extended to assess the effects and mechanisms of pharmaceutical toxicants and chemotherapy drugs on testis cell types, which are currently tested predominantly on rodent models^40–42^.

In conclusion, we demonstrate the responsiveness of primary human testicular cells to experimental perturbations by exogenous administration of a protein kinase inhibitor known to interfere with *in vitro* cord formation. We further propose our *in vitro* model as a functional platform to quantitatively investigate the processes of seminiferous tubulogenesis by pharmacological interference.

## Materials and Methods

### Testis tissue source and isolation of testicular cell suspensions

In total, 22 testes were procured from 22 adult gender dysphoria patients undergoing sex confirming surgery at the Urological Department of University Clinic of Essen, Germany. Ethical approval for the use of testicular tissue was obtained from the ethical committee of the Ärztekammer Westfalen-Lippe (no. 2012-555-f-S). Prior to surgery, patients had undergone gender confirming hormone therapy with anti-androgens (cyproterone acetate) and oestrogens. Clinical standard procedure was to discontinue cross-sex hormone therapy two weeks before surgery. We have previously published a detailed characterisation of our patient cohort regarding steroidogenesis status, testicular tissue histology and evaluation of degree of spermatogenesis via histological and flow cytometry analyses^16,43–46^. All experiments were performed in accordance with the relevant institutional guidelines and regulations. Testes were used after the patients provided written informed consent. Testes were transported in chilled Dulbecco’s modified Eagle’s medium low Glucose (1 g/l) + Pyruvate + L-Glutamine (31885; Gibco Life Technologies, USA) (DMEM) at 4 °C. Testes were decapsulated and 16 fragments of approx. 100 mg were dissected mechanically into 1–3 mm^3^ fragments with scissors. To obtain testicular cell suspensions, each sample was subjected individually to a two-step enzymatic digestion following a previously described protocol^16^. Each cell suspension that was generated from the 22 testes was considered an independent biological experiment.

### Cell culture conditions

Enzymatically generated testicular cell suspensions were seeded at density of 5×10^5^ cells/well in 24-well plates (83.3922; Sarstedt AG, Nümbrecht, Germany). Cells were cultured in DMEM, supplemented with 1% (v/v) MEM non-essential amino acids (11140; Gibco Invitrogen, Darmstadt, Germany), 1% (v/v) Anti/Anti (110X15240–062; Gibco Life Technologies, Grand Island, NY, USA). The medium for cultured cells analysed by downstream immunohistochemical analysis was additionally supplemented with 10% (v/v) KnockOut^TM^ serum replacement (10828010; Gibco, Life Technologies, USA). The commercially available protein kinase inhibitor K252a (K1639; Sigma, Steinheim, Germany), isolated from *Nonomuraea longicatena*, was applied at the time of cell plating. The compound was dissolved in dimethyl sulfoxide, DMSO (D5879; Sigma-Aldrich, Steinheim, Germany). Intermediate stocks of 10 µM, 4 µM, 2 µM, 1 µM, 0.1 µM and 0.01 µM were prepared by diluting the stock solution in culture medium. The final concentration range for K252a treatment groups that were attained in the wells containing plated testicular cells were 5 µM, 2 µM, 500 nM, 200 nM, 100 nM, 10 nM, 5 nM and 1 nM. For control experiments, cells were either cultured in culture medium alone (no treatment control) or DMSO was added to culture medium (vehicle control) in appropriate dilution (0.5% v/v) comparable to that in treatment groups. Cells were incubated at 35 °C and 5% CO_2_. Upon medium renewal 48 h after initial cell seeding^16^, the medium contained corresponding concentrations of K252a (1 nM–5 µM). Cells were then cultured for another 1–3 days before image acquisition for quantitative analysis. The experimental design was based on previously published studies exploring the effects of K252a within a 48–72-hour window of challenge^18–21,30^.

Additional cultures were exposed to vehicle control, 500 nM and 5 µM K252a for two weeks without medium renewal to account for the influence of high K252a concentrations on cellular re-aggregation over extended culture periods under constant exposure. Images were acquired on days 7 (n = 13) and 14 (n = 11) for qualitative analysis.

In a second set of experiments, K252a was introduced at cell seeding at a defined range of concentrations (100 nM–2 µM) for 48 h after seeding (withdrawal experiments) and was then replaced by culture medium only, which did not contain any K252a or DMSO. Cells were cultured for another 2-3 days when image acquisition for quantitative analysis was performed.

### Evaluation of cell viability

Testicular cells were cultured for 48 h under control conditions (no treatment or vehicle) and under exposure to 1 nM, 100 nM and 5 µM K252a. Cell viability was determined after 48 hours of single K252a challenge as this was the first time point when medium exchange was performed following our published protocol^16^. Medium containing the floating cell population was aspirated, pooled from the triplicates and pelleted at 536 g for 7 minutes. The pellet was resuspended in DMEM and live vs. dead cell numbers were determined via viability assessment with 0.4% (w/v) trypan blue. To enumerate live and dead cells in the adherent cell population, adherent cells were detached by incubation with 0.05% trypsin/EDTA for 1 minute at room temperature. Reaction was stopped by adding equal volume of DMEM to each well. Proportion of all live cells (in floating and adherent fraction) expressed out of total number of live and dead cells (in floating and attached fraction) was calculated. Accordingly, percentage live cells attached, expressed as the proportion of live cells in the attached fraction out of the total number of live cells (in floating and attached cell fraction) was evaluated. Each condition was performed in technical triplicates and in at least five independent experiments per condition.

### Immunohistochemical staining of cross-sections and whole cord-like structures formed *in vitro*

For detection of marker proteins of Sertoli and peritubular cells, a double staining for SRY-related high mobility box 9 (SOX9) and alpha-smooth muscle actin (αSMA) was performed on fixed aggregates whole and cross-sections of paraffin embedded cord-like structures^47^. The following antibodies were used: polyclonal rabbit anti-SOX9 (Dilution 1:100; AB5535 Millipore), monoclonal mouse anti-αSMA (Dilution 1:500 or 1:1000; A2547; Sigma-Aldrich, Germany). SOX9 antibody was visualized by secondary horseradish peroxidase-labelled chicken anti-rabbit IgG (Dilution 1:100; SC-2955, Santa Cruz Biotechnology, Paso Robles, CA, USA). Diaminobenzidine (DAB), used as a substrate, generated a brown signal at immunopositive sites. Alpha-SMA was visualized by secondary alkaline phosphatase-labelled chicken anti- mouse IgG (Dilution 1:100; SC-2958, Santa Cruz Biotechnology, Paso Robles, CA, USA). The use of DAKO REAL detection system alkaline phosphatase (K5005; DAKO, Denmark) produced a red precipitate in immunopositive regions. For SOX9 and αSMA staining, a previously described protocol was used^16^. Briefly, whole aggregates and aggregate cross-sections were washed twice with distilled water before blocking of non-specific binding by incubation with 5% chicken serum diluted in 0.1% bovine serum albumin, BSA. Samples were incubated overnight at 4 °C with a mix of SOX9 and αSMA antibodies, both diluted in 0.1% BSA. Incubation with secondary antibodies was performed on the next day after several washing steps, followed by further washing steps before development of colour reaction with DAB for 4–6 minutes. Afterwards, DAKO REAL detection system for 2–3 minutes was applied, interrupted by washing steps with Tris-buffered saline (TBS) in between. Samples were counterstained with haematoxylin (Mayer’s hemalaum solution; 1092490500; Merck Millipore, Darmstadt, Germany) and mounted in Faramount (S3025; DAKO, Glostrup, Denmark).

Single staining for tight junction protein Zonula occludens-1 (ZO-1) on cross-sections of paraffin embedded cord-like structures followed the same protocol as previously described^48^. Polyclonal rabbit anti-ZO-1 antibody (Dilution 1:100; 61–7300; ThermoFisher, Germany) was visualized by secondary chicken anti-rabbit IgG-Biotin antibody (Dilution 1:200; SAB3700943-1; Sigma-Aldrich, Germany) followed by streptavidin-horse-radish peroxidase (Dilution 1:500; s5512; Sigma-Aldrich, Germany) and DAB was used as a substrate. Primary and secondary antibodies and streptavidin-horseradish peroxidase were diluted in 25% chicken serum in 0.5% BSA in TBS.

### Micrograph acquisition and time-lapse imaging

Phase-contrast micrographs for qualitative and quantitative analyses were acquired with an inverted microscope (CKX41, Olympus, Melville, NY, USA) equipped with CellSense software (v. 1.5; Olympus, 2010, Melville, NY, USA) and with Zeiss Observer Z1 microscope (Carl Zeiss Microscopy, Röttingen, Germany) equipped with Axio vision (v. 4.8.2.0; Axio Vs40, Carl Zeiss MicroImaging) or Zen blue (v. 2.3 pro; Carl Zeiss Microscopy, Jena, Germany) software.

For live-cell imaging, testicular cell suspensions were seeded at a density of 5 × 10^5^ cells/well on 24-well plates (3526; Costar-Corning, NY, USA) under constant exposure to 500 nM (n = 7) and 5 µM (n = 7) K252a. No treatment (n = 6) and vehicle controls (n = 5) were run in parallel. During the experiments, plates were kept in a live cell imaging incubator (PMS1; 160-800 006; PeCon) with a heated insert (M24 S1; 130–800 015; PeCon) at 35 °C and 5% CO_2_. Images (referred to as frames) were captured every 10 minutes over a period of 16–44 hours with a Zeiss Observer Z1 microscope (Carl Zeiss Microscopy, Röttingen, Germany) equipped with Zeiss AxioCam Mrm camera (Carl Zeiss MicroImaging, Röttingen, Germany) and Axio vision software (v. 4.8.2.0; AxioVs40, Carl Zeiss MicroImaging) or with AxioCam 506 mono camera (Carl Zeiss MicroImaging, Röttingen, Germany) and Zen blue software (v. 2.3 pro; Carl Zeiss Microscopy, Jena, Germany) at 10x magnification. Videos are presented as 5 frames/second. One frame in the video corresponds to 10 minutes real-time. Data processing was performed with open source Fiji software (http://fiji.sc/), Adobe Photoshop CC (v. 2017.0.1; Adobe Systems Inc., San Jose, CA, USA) and Adobe InDesign CC (v.2017.0; Adobe Systems Inc., San Jose, CA, USA).

### Image acquisition for quantitative analysis

Phase-contrast micrographs of testicular cell suspensions treated with K252a under constant exposure (1 nM n = 10; 5 nM n = 9; 10 nM n = 9; 100 nM n = 10; 200 nM n = 8; 500 nM n = 19; 2 µM n = 8; 5 µM n = 14; vehicle n = 19; no treatment n = 9) or in withdrawal experiments (100 nM–2 µM n = 7; vehicle n = 7) were captured at 10x magnification 3–5 days after cell seeding. Image acquisition was initiated when aggregate compaction in controls was observed. Each condition was analysed in triplicates. A random systematic sampling strategy was applied for image acquisition. Each replicate well was optically divided in quadrants and four micrographs were captured from four randomly selected fields in each quadrant. Overall, 16 micrographs were acquired per replicate (well), hence 48 micrographs in total from three replicates per condition.

### Image processing and digital data generation

**Figure 6:**
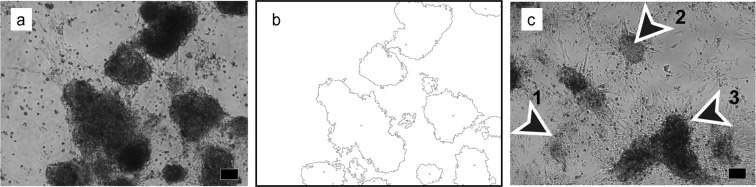
Raw data processing in Fiji software for quantitative analysis (**a**,**b**) and representative phase-contrast image showed three aggregates assigned to three size categories that were used to assess the effect of K252a on cellular reassembly. Representative phase-contrast micrograph (**a**) before importing in Fiji for processing, and a summary image (**b**) after processing following importing in Fiji and analysing with macro instructions. Note that after subjecting the micrograph to the tailored macro instructions to retrieve data for aggregate size, the information in the summary image was corresponding to the original phase-contrast micrograph (**b**). (**c**) Phase-contrast image showing aggregates representing three out of five size categories used to describe the effect of K252a on testicular cell reassembly– arrowhead 1: 1500–3000 µm^2^, arrowhead 2: 6000–12000 µm^2^, arrowhead 3: >20000 µm^2^. Scale bars = 100 µm.

All 48 micrographs for each condition within an experiment were processed with Fiji software^49^. A sequence of macro instructions was created for automatic quantitative analysis. After a flat field correction, thresholding was used to generate a binary mask of the aggregate boarders. This mask was further processed to smooth the outline of the aggregates and fill small holes without changing the overall size (Fig. 6a,b). These aggregates were analysed with the “analyse particle” routine in Fiji.

The intra-assay coefficient of variation, CV, (n = 5) was used as a measure of the validity of the data acquisition approach and the macro instructions created in Fiji. It also served to confirm the reproducibility of the established culture approach. A mean of individual aggregate size was calculated for each of the 16 images taken per replicate and the total number of aggregates per image was recorded. Then, a mean value was calculated out of the 16 summary values. These three values were used to calculate the intra-assay CV for each individual experiment.

Data about aggregate number and size from treatment conditions and controls were subjected to the same processing algorithm as for intra-assay CV. Arithmetic means calculated from the three values for controls and treatment conditions were used for statistical analysis. To calculate IC50 for the parameter aggregate size, the aggregate size for each set of experiments was normalized to the aggregate size of the vehicle control. The mean relative aggregate sizes were plotted against the K252a concentration and fitted with a logistic function using Origin (OrginLab 2019).

Additionally, all values for aggregate size from the raw datasets, generated from 16 micrographs/replicate in each condition from 9 independent experiments (treatment control, vehicle control, 500 nM K252a, 100 nM K252a, 10 nM K252a, 5 nM K252a and 1 nM K252a) and 4 independent experiments (5 µM K252a), were plotted in GraphPad Prism 5 (v. 5; GraphPad Software, La Jolla, CA, USA) to define data distribution. Data distribution was used to define 5 size categories (1500–3000 µm^2^, 3000–6000 µm^2^, 6000–12000 µm^2^, 12000–20000 µm^2^, >20000 µm^2^; Fig. 1c and Supplementary Fig. 1), based on the observation that 90% of data points were <20000 µm^2^ (Supplementary Fig. 1). Then, aggregate number in each size category was calculated for each condition: constant exposure experimental series: 1 nM (n = 10), 5 nM (n = 9), 10 nM (n = 9), 100 nM (n = 10), 200 nM (n = 8), 500 nM (n = 19), 2 µM (n = 8), 5 µM (n = 14), vehicle (n = 19), no treatment (n = 9) and withdrawal experimental series – 100 nM–2 µM (n = 7), vehicle (n = 7).

### Statistical analysis

Data were tested for normality via D’Agostino and Pearson omnibus normality test. Mann-Whitney U, Kruskal-Wallis and one-way ANOVA tests were applied depending on data distribution. Percentage of all live cells and percentage of live cells attached, aggregate size, aggregate number and aggregate number in size categories were statistically analysed, using GraphPad Prism 5 (v. 5) or 8 (v.8.2.0) software as stated in the figure legends. In each experimental set-up, no treatment and vehicle controls were statistically compared and, as they were not different, statistical comparison was further performed between treatment conditions and vehicle control. Percentage all live cells and percentage live cells attached from treatment groups were compared to no treatment control. A p value < 0.05 was considered significant.

## Supplementary information

Suppl Figure 1 and 2 combined.

Supplementary Legens.

Suppl Video 1.

Suppl Video 2.

Suppl Video 3.

Suppl Video 4.
