# Supplementary material for: Challenging human somatic testicular cell reassembly by protein kinase inhibition –setting up a functional in vitro test system
Source: Sci Rep. 2020 Jun 2;10:8935. doi: 10.1038/s41598-020-65924-y (PMC7265505; doi:10.1038/s41598-020-65924-y)

SREP-19-38588
Challenging human somatic testicular cell reassembly by protein kinase inhibition –setting up a functional in vitro test system
M. Mincheva, J. Wistuba, C. Brenker, S. Schlatt

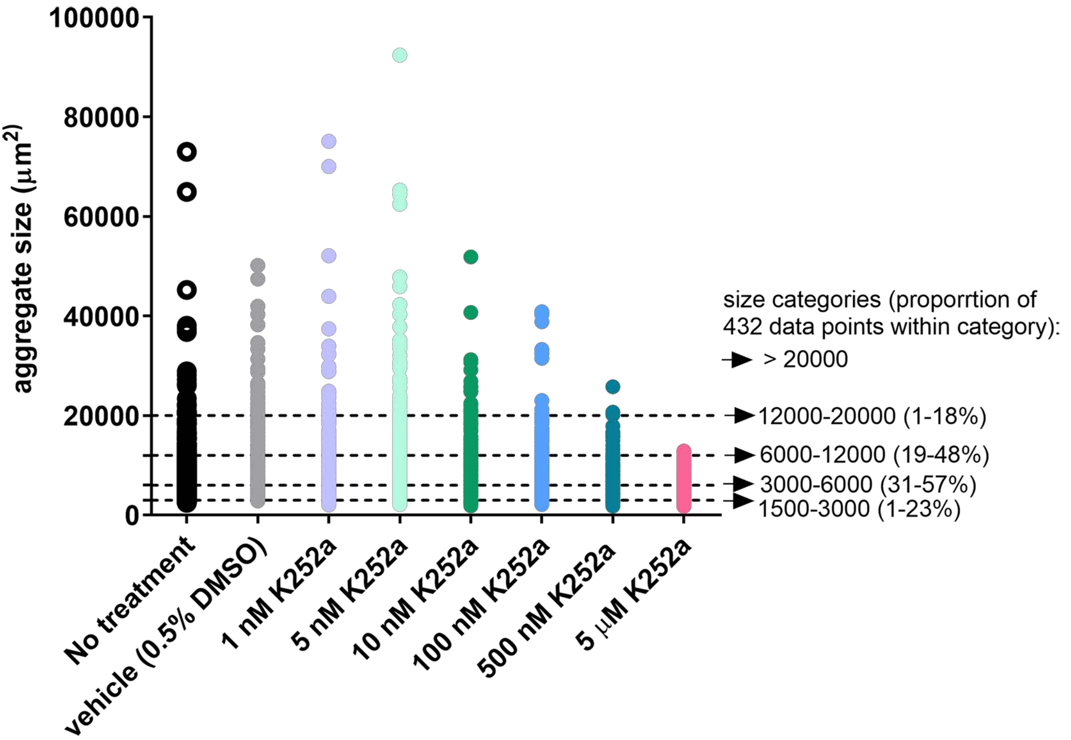

SREP-19-38588
Challenging human somatic testicular cell reassembly by protein kinase inhibition –setting up a functional in vitro test system
M. Mincheva, J. Wistuba, C. Brenker, S. Schlatt

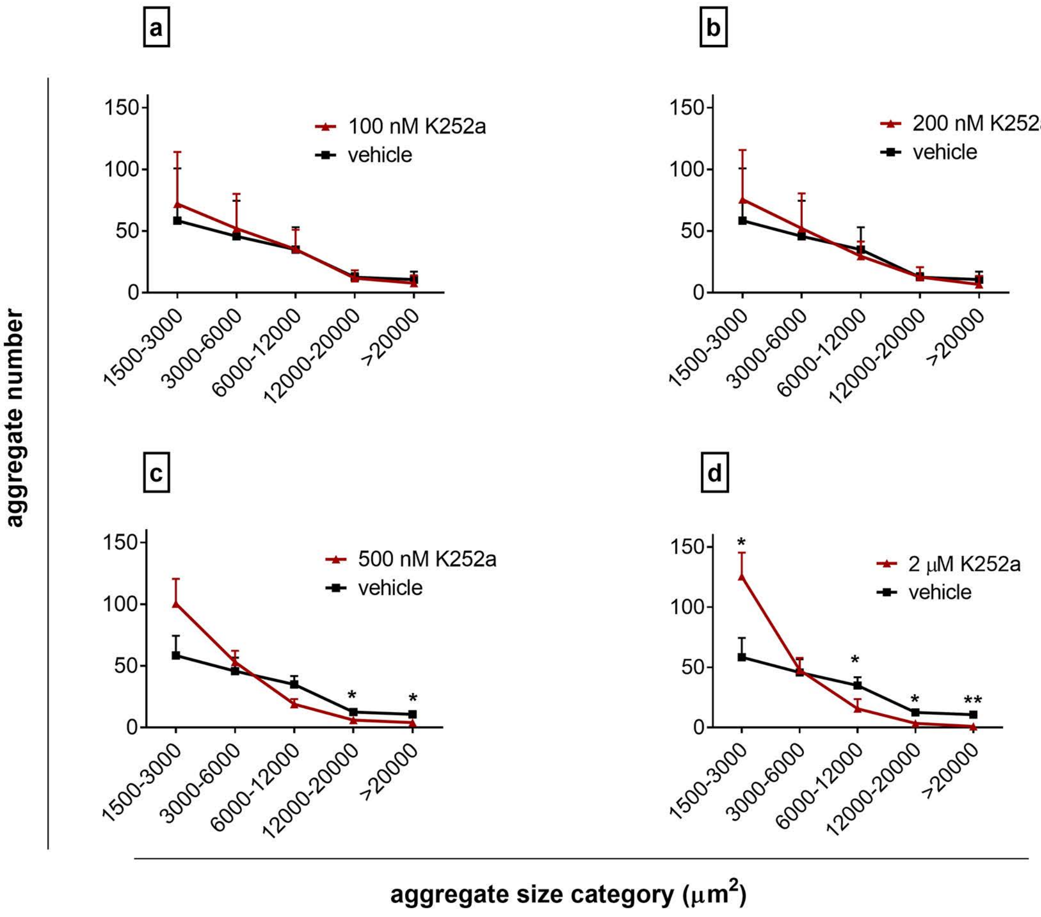

Supplement: Supplementary file 1 — Suppl Figure 1 and 2 combined. [file 41598_2020_65924_MOESM1_ESM.pdf]
