# Supplementary material for: Challenging human somatic testicular cell reassembly by protein kinase inhibition –setting up a functional in vitro test system
Source: Sci Rep. 2020 Jun 2;10:8935. doi: 10.1038/s41598-020-65924-y (PMC7265505; doi:10.1038/s41598-020-65924-y)
Supplement: Supplementary file 2 — Supplementary Legens. [file 41598_2020_65924_MOESM2_ESM.docx]

**Supplementary Material Legend**

**Supplementary Figure 1 Visualisation of raw data distribution for the parameter 'aggregate size' in K252a treated and control groups used to assign 5 aggregate size categories.**

Datasets from 9 experiments (for treatment control, vehicle control, 500 nM K252a, 100 nM K252a, 10 nM K252a, 5 nM K252a and 1 nM K252a) and from 4 experiments (for 5 µM K252a) were combined and plotted together to define data distribution. Each line in no treatment control, vehicle control, 500 nM K252a, 100 nM K252a, 10 nM K252a, 5 nM K252a and 1 nM K252a presents 432 data points and 192 data points in 5 µM K252a. Individual data point represents median aggregate size calculated for each of the 48 micrographs in every experiment taken for each treatment group. Dotted lines mark aggregate size categories assigned based on data distribution. Percentage of data points falling within each size category is indicated in brackets.

**Supplementary Figure 2 Short-term exposure to K252a (withdrawal experiments) affect number size across size categories**

Human testicular cells were cultured for 2 days under exposure to K252a at 200 nM – 2 µM, followed by wash-out and culture for another 2-3 days in culture medium only (without any K252a or 0.5% DMSO). Two-day exposure to K252a at 2 µM and 500 nM resulted in decreased number of aggregates above 12000 µm^2^ (at 500 nM) and 6000 µm^2^ (at 2 µM) of size (c and d, respectively). Two-day incubation with 2 µM K252a increased aggregate numbers in the smallest size category (d). Data are shown as mean (+SEM), Mann-Whitney U test versus vehicle control, *p < 0.05, **p < 0.01.

**Supplementary Video 1 Time-lapse video recording revealing the processes of testicular cord formation in no treatment control**

Testicular cells re-assemble in round aggregates, which compact further and coalesce in round cords. Approximately, 5 x 10^5^ cells / well were seeded. Recording was started on day two after seeding and lasted for approximately for 16h, with one record every 10 minutes. In total, 145 frames were recorded and converted in a video with duration of 28 seconds with 5 frames per second. One frame in the video corresponds to 10 minutes real-time, i.e. one second in the video equals 50 minutes real time.

**Supplementary Video 2 Time-lapse video recording revealing aggregate compaction and initiation of aggregate interconnection in vehicle control**

Approximately, 5 x 10^5^ cells / well were seeded. Recording was started on day two after seeding and lasted for approximately for 44h, with one record every 10 minutes. In total, 263 frames were recorded and converted in a video with duration of 52 seconds with 5 frames per second. One frame in the video corresponds to 10 minutes real-time, i.e. one second in the video equals 50 minutes real time.

**Supplementary Video 3 Time-lapse video recording revealing the effect of 500 nM K252a on cellular movements.** Elongated cells protrude radially from the aggregates, but no aggregate coalescence takes place

Approximately, 5 x 10^5^ cells / well were seeded. Recording was started on day two after seeding and lasted for approximately for 44h, with one record every 10 minutes. In total, 263 frames were recorded and converted in a video with duration of 52 seconds with 5 frames per second. One frame in the video corresponds to 10 minutes real-time, i.e. one second in the video equals 50 minutes real time.

**Supplementary Video 4 Time-lapse video recording revealing no cellular movements towards aggregates formed under exposure to 5 µM K252a.** Aggregates remain still with smooth edges and no elongated cells protrude from them

Approximately, 5 x 10^5^ cells / well were seeded. Recording was started on day two after seeding and lasted for approximately for 44h, with one record every 10 minutes. In total, 263 frames were recorded and converted in a video with duration of 52 seconds with 5 frames per second. One frame in the video corresponds to 10 minutes real-time, i.e. one second in the video equals 50 minutes real time.
